# Surfactant protein A genetic variants associate with severe respiratory insufficiency in pandemic influenza A virus infection

**DOI:** 10.1186/cc13934

**Published:** 2014-06-20

**Authors:** Estefanía Herrera-Ramos, Marta López-Rodríguez, José Juan Ruíz-Hernández, Juan Pablo Horcajada, Luis Borderías, Elisabeth Lerma, José Blanquer, María Carmen Pérez-González, María Isabel García-Laorden, Yanira Florido, Virginia Mas-Bosch, Milagro Montero, José María Ferrer, Luisa Sorlí, Carlos Vilaplana, Olga Rajas, Marisa Briones, Javier Aspa, Eduardo López-Granados, Jordi Solé-Violán, Felipe Rodríguez de Castro, Carlos Rodríguez-Gallego

**Affiliations:** 1 Department of Immunology, Hospital Universitario de Gran Canaria Dr. Negrín, Las Palmas de Gran Canaria 35010, Spain; 2 Department of Medical and Surgical Sciences, School of Medicine, Universidad de Las Palmas de Gran Canaria, Las Palmas de Gran Canaria 35016, Spain; 3 Department of Internal Medicine, Hospital Universitario de Gran Canaria Dr Negrín, Las Palmas de Gran Canaria 35010, Spain; 4 Department of Infectious Diseases, Hospital Universitari del Mar, Barcelona 08003, Spain; 5 Hospital del Mar de Investigaciones Médicas (IMIM), CIBERES, Barcelona 08003, Spain; 6 Department of Respiratory Diseases, Hospital San Jorge, Huesca 22004, Spain; 7 Intensive Care Unit, Hospital Clínico y Universitario de Valencia, Valencia 46010, Spain; 8 Department of Microbiology, Hospital Universitario de Gran Canaria Dr. Negrín, Las Palmas de Gran Canaria 35010, Spain; 9 Center for Experimental and Molecular Medicine, Academic Medical Center, Amsterdam 1105 AZ, The Netherlands; 10 Laboratori de Referència de Catalunya, Prat de Llobregat, Barcelona 08820, Spain; 11 Intensive Care Unit, Hospital Universitario de Gran Canaria Dr. Negrín, CIBERES, Las Palmas de Gran Canaria 35010, Spain; 12 Department of Respiratory Diseases, Hospital Universitario de la Princesa, Madrid 28005, Spain; 13 Department of Respiratory Diseases, Hospital Clínico y Universitario de Valencia, Valencia 46010, Spain; 14 Department of Immunology, Hospital La Paz, Madrid 28046, Spain; 15 Department of Respiratory Diseases, Hospital Universitario de Gran Canaria Dr. Negrín, Las Palmas de Gran Canaria 35010, Spain

## Abstract

**Introduction:**

Inherited variability in host immune responses influences susceptibility and outcome of Influenza A virus (IAV) infection, but these factors remain largely unknown. Components of the innate immune response may be crucial in the first days of the infection. The collectins surfactant protein (SP)-A1, -A2, and -D and mannose-binding lectin (MBL) neutralize IAV infectivity, although only SP-A2 can establish an efficient neutralization of poorly glycosylated pandemic IAV strains.

**Methods:**

We studied the role of polymorphic variants at the genes of MBL (*MBL2*), SP-A1 (*SFTPA1*), SP-A2 (*SFTPA2*), and SP-D (*SFTPD*) in 93 patients with H1N1 pandemic 2009 (H1N1pdm) infection.

**Results:**

Multivariate analysis showed that two frequent *SFTPA2* missense alleles (rs1965708-*C* and rs1059046-*A*) and the *SFTPA2* haplotype *1A*^*0*^ were associated with a need for mechanical ventilation, acute respiratory failure, and acute respiratory distress syndrome. The *SFTPA2* haplotype *1A*^*1*^ was a protective variant. Kaplan-Meier analysis and Cox regression also showed that diplotypes not containing the *1A*^*1*^ haplotype were associated with a significantly shorter time to ICU admission in hospitalized patients. In addition, rs1965708-*C* (*P* = 0.0007), rs1059046-*A* (*P* = 0.0007), and haplotype *1A*^*0*^ (*P* = 0.0004) were associated, in a dose-dependent fashion, with lower PaO_2_/FiO_2_ ratio, whereas haplotype *1A*^*1*^ was associated with a higher PaO_2_/FiO_2_ ratio (*P* = 0.001).

**Conclusions:**

Our data suggest an effect of genetic variants of *SFTPA2* on the severity of H1N1pdm infection and could pave the way for a potential treatment with haplotype-specific (*1A*^*1*^) SP-A2 for future IAV pandemics.

## Introduction

Influenza A virus (IAV) infection is usually a mild and self-limited condition. Likewise, infection with the H1N1 pandemic 2009 (H1N1pdm) IAV often results in an uncomplicated flu, but, in a small subset of patients, it may rapidly evolve to primary viral pneumonia, acute respiratory failure (ARF), and acute respiratory distress syndrome (ARDS) [1]. Inherited and acquired variability in host immune responses may influence susceptibility and outcome of IAV infection, although these factors remain largely unknown [2-4].

Before exposure to H1N1pdm IAV, most individuals, particularly those born after 1957, lack serum antibodies capable of neutralizing the virus [1]. Adaptive immune responses, which are needed for ultimate viral clearance, take several days to develop. Therefore, components of the innate immunity that are able to neutralize IAV infection with minor inflammation may be crucial for host defense in the first few days after infection. Different soluble pattern-recognition molecules of the innate immunity can neutralize IAV infection. Several secreted human C-type lectins of the collectin family, the serum mannose-binding lectin (MBL), the pulmonary surfactant proteins (SP) –A1, –A2, and –D, and collectin 11 (CL-11, alias collectin kidney 1, CL-K1) may neutralize IAV infectivity *in vitro*[5-8]. Among these collectins, the SPs have been shown to exert an important role against IAV infection in animal models: mice lacking SP-A or SP-D have increased susceptibility to IAV infection, and their role seem to depend on IAV strains, particularly pandemic versus seasonal strains [9-14]. In addition, variability at the collectin genes, *MBL2* (Ensembl: ENSG00000165471), *SFTPA1* (Ensembl: ENSG00000122852), *SFTPA2* (Ensembl: ENSG00000185303), and *SFTPD* (Ensembl: ENSG00000133661) have been found to be associated with susceptibility to and/or severity of several bacterial and viral infectious diseases [6,15].

It hence follows that these collectins are firm candidates to explain, at least in part, the role of host genetic variability in the defense against IAV infection. The human SP-A locus consists of two similar genes, *SFTPA1* and *SFTPA2*, localized within a cluster (10q21-24) that includes the SP-D gene (*SFTPD*) [16]. *MBL2* was reported not to be in physical linkage with the genes of these SPs [17], but linkage disequilibrium of *MBL2* with *SFTPA1* and *SFTPA2* has been shown; and LD among variants at these genes could influence the results of genetic-association studies [6,18,19].

In the present study, we assessed the role of variants at the *SFTPA1*, *SFTPA2,* and *SFTPD* genes in H1N1pdm IAV infection in humans. Variants at the neighbor collectin gene *MBL2* were also analyzed.

## Materials and methods

### H1N1pdm-infected patients

**Figure 1:**
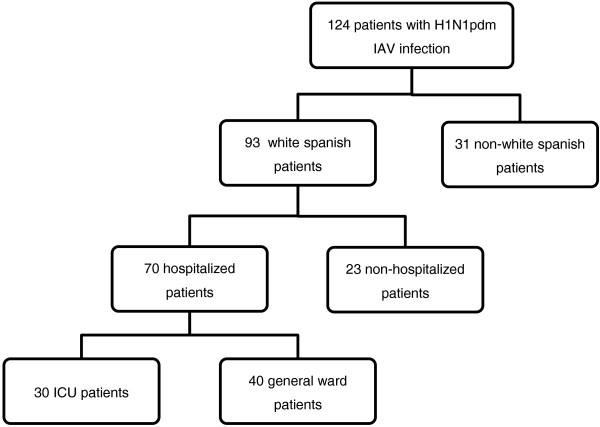
**Selection process for patients with H1N1pdm 2009 infection.** Infection by H1N1pdm was confirmed in all the 124 patients.

We recruited 124 patients with H1N1pdm infection between July 2009 and November 2011. Thirty-one individuals with ancestors other than Spanish were excluded. Of 93 unrelated white Spanish patients, 70 were hospitalized at five tertiary Spanish hospitals, and 23 were attended at primary care centers (Figure 1). Data and samples from all ambulatory patients and from 30% of hospitalized individuals were retrospectively obtained; in the remaining patients, data were obtained prospectively. All patients were treated with oseltamivir, and only one patient had been previously vaccinated against H1N1pdm.

Diabetes, previous lung disease, solid-organ transplantation, immunosuppression, body mass index (BMI) ≥30, human immunodeficiency virus (HIV) infection, and pregnancy were considered risk factors. Sepsis, septic shock, and multiorgan dysfunction syndrome (MODS) were defined by using the American College of Chest Physicians/Society of Critical Care Medicine criteria [20]. ARF and ARDS were diagnosed according to the American European Consensus Conference Definition [21]. Functional parameters of gas exchange were calculated on the basis of the ratio of oxygen arterial pressure to oxygen inspiratory fraction (PaO_2_/FiO_2_). In hospitalized patients without arterial blood gas analysis (*n* = 18), ARF was established when hemoglobin oxygen saturation, breathing room air, was lower than 90%. In patients with intensive care unit (ICU) admission, severity of illness was evaluated with the Acute Physiology and Chronic Health Evaluation II score, taking the worst reading of the first 24 hours in the ICU.

All steps were performed in complete accordance to the Helsinki declaration. The protocol was approved by Clinical Research Ethics Committees (CEIC) of hospitals involved (CEIC Hospital Universitario de Gran Canaria Doctor Negrín, Las Palmas de Gran Canaria; CEIC Hospital San Jorge, Huesca and CEIC Hospital Clínico y Universitario de Valencia, Valencia). Informed consent was obtained of all patients included.

### Detection of H1N1pdm by real-time polymerase chain reaction

Influenza A H1N1 virus in the 124 patients was detected in nasopharyngeal swabs by using the Real-Time ready Influenza A (H1N1) Detection Set (Roche Diagnostics GmbH, Mannheim, Germany) according to manufacturer’s protocol.

### General Spanish-population subjects

The general population group consisted of white unrelated Spanish healthy volunteers (blood and bone marrow donors as well as hospital staff) from four tertiary Spanish hospitals. The control group was analyzed for *MBL2* (*n* = 1,736) and *SFTPA1* and *SFTPA2* (*n* = 769) polymorphisms in previous studies [18,19]. For the nine *SFTPD* variants under study, 963 individuals from the general population group recruited at the same hospitals were analyzed in this study. The protocol was approved by Clinical Research Ethics Committees (CEIC) of hospitals involved (CEIC Hospital Universitario de Gran Canaria Doctor Negrín, Las Palmas de Gran Canaria; CEIC Hospital de la Princesa, Madrid; CEIC Hospital San Jorge, Huesca and CEIC Hospital Clínico y Universitario de Valencia, Valencia). Informed consent was obtained of the general-population individuals included. No data about whether individuals from the general Spanish population group were or were not infected with H1N1pdm were available. Foreigners and individuals with ancestors other than Spanish were previously excluded.

### Selection of single-nucleotide polymorphisms

Deficient and low MBL serum levels are mainly due to the presence of three common point mutations in the exon 1 of the *MBL2* gene: alleles *B* (rs1800450), *C* (rs1800451), and *D* (rs5030737) are termed *O* alleles, *A* being the wild-type. Serum MBL levels are very low or absent in individuals homozygous for *O* alleles. In addition, the presence of the promoter allele *X* (rs7096206) has an important downregulating effect, and individuals with *XA*/*O* also have very low MBL serum levels. *O*/*O* together with *XA*/*O* genotypes are considered MBL-deficient genotypes, which are common in most populations.

The human SP-A locus consists of two similar genes, *SFTPA1* and *SFTPA2*, located on chromosome 10q22.3, within a cluster that includes the SP-D gene (*SFTPD*). In addition, a certain degree of linkage disequilibrium (LD) exists among SP genes and the MBL gene (10q21-24) [18,19]. On the basis of the existence of several SNPs, SP-A haplotypes are conventionally denoted as *6A*^*n*^ for the *SFTPA1* gene (V19A, rs1059047; L50V, rs1136450; R219W, rs4253527) and *1A*^*n*^ for the *SFTPA2* gene (T9N, rs1059046; A91P, rs17886395; Q223K, rs1965708) [22]. These missense single-nucleotide polymorphisms (SNPs) at *SFTPA1* and *SFTPA2* were analyzed in our study. The most frequent conventional haplotypes of these genes, except *1A* and *1A*^*5*^, can be unambiguously identified.

For the analysis of *SFTPD*, genotypic data of individuals of European ancestry (CEU) from the International HapMap Project [23] were used to select LD tagging SNPs. Pairwise LD-tagging was achieved with Haploview v. 4.2 [24] for SNPs with a minimum minor allele frequency of 0.05 and *r*^*2*^ of 0.8. As result, three intronic SNPs (rs10887199, rs7078012, and rs723192) and one synonymous SNP (rs6413520) were selected. Three missense SNPs were also analyzed: rs3088308 (S290T), rs2243639 (T180A), and rs721917 (M31T). Additionally, we studied an SNP in the *SFTPD* promoter region (rs1885551) that was recently reported to have a profound impact on serum SP-D levels [25] and an intronic SNP (rs17886286) associated with susceptibility to invasive pneumococcal disease [26].

### DNA extraction and genotyping

Genomic DNA was extracted from 400 μl of peripheral blood by using iPrep PureLink gDNA Blood kit in the iPrep Purification Instrument (Invitrogen by Life Technologies, Carlsbad, CA, USA). Extracted DNA integrity was checked by NanoDrop ND-1000 (NanoDrop Technologies, Wilmington, DE, USA). In total, 19 polymorphisms at *MBL2*, *SFTPA1*, *SFTPA2* and *SFTPD* were studied.

*MBL2, SFTPA1, SFTPA2,* and *SFTPD* rs721917 SNPs were analyzed by means of PCR-RFLP and PCR-SSP techniques, as previously described [18,19]. The other eight SNPs of *SFTPD* were determined by predesigned Taqman SNP genotyping assays (Assays IDs: C__26726209_10, C__26726205_10, C__31530298_10, C__63652102_10, C__29213175_10, C___1362981_20, C____630297_10, and C__12124527_20), according to the manufacturer’s protocol, with commercially available reagents by means of ViiA™7 Real-time PCR System (Applied Biosystems, Foster City, CA, USA).

### Data analysis

The Hardy-Weinberg equilibrium was analyzed with Haploview v. 4.2. Haplotypes were estimated *in silico* with PHASE v. 2.1.1 under 1,000 permutations. Statistical analyses were performed by using SPSS 20.0 (SPSS, Inc., Chicago, IL, USA). The comparison of all genotype distributions based on susceptibility and severity was performed by using the χ^2^ test or Fisher Exact test when needed, and odds ratios with 95% confidence intervals were calculated. The relation between severity in hospitalized patients and genotypes was evaluated by binary logistic regression models: age, gender, risk factors, and secondary infection (either bacteremia or secondary bacterial pneumonia) were included as independent variables.

Comparison of the PaO_2_/FiO_2_ ratio according to genetic variants was performed by a using linear regression model adjusted by age, gender, risk factors, and secondary infection (either bacteremia or secondary bacterial pneumonia).

To assess the need for ICU admission, a multivariate analysis was performed, including the aforementioned variables, by conditional logistic regression stratified by hospital of origin.

Log-rank (LR) χ^2^ tests were calculated to compare ICU admission according to the distribution of genetic variants. Cox proportional hazard ratios adjusted for the independent variables age, gender, risk factors, development of secondary bacterial infection (pneumonia or bacteremia), and hospital of origin were also performed.

## Results

Demographic and clinical characteristics of patients are shown in Table 1. The genotype distribution of SNPs did not differ significantly under conditions of Hardy-Weinberg equilibrium in all of the groups studied (data not shown). Frequencies of the genetic variants under analysis were not found to be significantly different between H1N1pdm-infected patients and the general population (Table 2).

**Table 1:** Demographic and clinical characteristics of H1N1pdm-infected patients

**Characteristics**	**Subjects**	
Age (years)	43.5 ± 18.0^a^	
Gender (male)	52	(55.9)
Hospital admission		
No	23	(24.7)
Yes	70	(75.3)
PVP		
No	40	(43.0)
Yes	53	(57.0)
ICU admission		
No	63	(67.7)
Yes	30	(32.3)
ARF		
No	45	(48.4)
Yes	48	(51.6)
Shock		
No	77	(82.8)
Yes	16	(17.2)
ARDS		
No	77	(82.8)
Yes	16	(17.2)
MODS		
No	87	(93.5)
Yes	6	(0.65)
MV		
No	69	(74.2)
Yes	24	(25.8)
Hospital mortality		
No	89	(95.7)
Yes	4	(0.43)
Risk factor		
No	32	(34.4)
Yes	61	(65.6)
Secondary bacterial infection^b^		
No	80	(86.0)
Yes	13	(14.0)

ARDS, acute respiratory distress syndrome; ARF, acute respiratory failure; ICU, intensive care unit; MODS, multiorgan dysfunction syndrome; MV, mechanical ventilation; PVP, Primary viral pneumonia.

^a^Data are presented as mean ± SD or number of individuals (%). ^b^Ten patients had secondary bacterial pneumonia, and seven patients had bacteremia (four of them with secondary bacterial pneumonia).

**Table 2:** Distribution of genotype frequencies of collectin genes in general Spanish population and H1N1pdm-infected patients

**Variants**	**Genotypes**	**General Spanish population**	**H1N1pdm-infected patients**
***MBL2***^**a**^		***n*** **= 1736**	***n*** **= 93**
rs1800451 (G57E)			
rs1800450 (G54D)	AA/AO/OO	1,032 (59.4)/615(35.4)/89 (5.1)	54 (58.1)/36 (38.7)/3 (3.2)
rs5030737 (R52C)			
rs7096206 (Prom)			
MBL deficiency	*AA* + *YAO*/*XAO* + *OO*	1,475 (85.0)/261 (15,0)	77 (82.8)/16 (17.2)
***SFTPA2***		***n*** **= 769**	***n*** **= 93**
rs1965708 (Q223K)	AA/CA/CC	22 (2.9)/244 (31.7)/503 (65.4)	4 (4.3)/33 (35.5)/56 (60.2)
rs17886395 (A91P)	GG/GC/CC	623 (81.0)/134 (17.4)/12 (1.6)	72 (77.4)/19 (20.4)/2(2.2)
rs1059046 (T9N)	CC/AC/AA	97 (12.,6)/349 (45.4)/323 (42.0)	14 (15.1)/48 (51.6)/31 (33.3)
***SFTPA1***		***n*** **= 769**	***n*** **= 93**
rs1059047 (V19A)	TT/TC/CC	680 (88.4)/88 (11.4)/1 (0.1)	82 (88.2)/10 (10.8)/1 (1.1)
rs1136450 (L50V)	CC/GC/GG	117 (15.2)/334 (43.4)/318 (41.4)	12 (12.9)/48 (51.6)/33 (35.5)
rs4253527 (R219W)	CC/CT/TT	620 (80.6)/142 (18.5)/7 (0.9)	76 (81.7)/16 (17.2)/1 (1.1)
***SFTPD***		***n*** **= 963**	***n*** **= 93**
rs3088308 (S290T)	AA/AT/TT	829 (86.1)/129 (13.4)/5 (0.5)	77 (82.8)/14 (15.1)/2 (2.1)
rs2243639 (T180A)	TT/TC/CC	373 (38.7)/449 (46.6)/141 (14.6)	42 (45.2)/42 (45.2)/9 (9.6)
rs10887199 (Intr)	TT/TC/CC	759 (78.8)/189 (19.6)/15 (1.6)	71 (76.3)/20 (21.5)/2 (2.2)
rs17886286 (Intr)	CC/CG/G	828 (86.0)/126 (13.1)/9 (0.9)	79 (85.0)/13 (14.0)/1 (1.0)
rs7078012 (Intr)	CC/CT/TT	629 (65.3)/292 (30.3)/42 (4.4)	52 (55,.)/38 (40.9)/3(3.2)
rs6413520 (S45S)	AA/AG/GG	824 (85.6)134 (13.9)/5 (0.5)	82 (88.2)/11 (11.8)/0 (0.0)
rs721917 (M31T)	TT/TC/CC	356 (37.0)/438 (45.5)/169 (17.5)	36 (38/.7)/38 (40.9)/19 (20.4)
rs723192 (Intr)	CC/CT/TT	757 (78.6)/195 (20.3)/11 (1.1)	70 (75.3)/21 (22.6)/2 (2.1)
rs1885551 (Prom)	AA/AG/GG	766 (79.5)/182 (18.9)/15 (1.6)	72 (77.4)/19 (20.4)/2 (2.2)

^a^*O/O* together with *XA/O* genotypes are considered MBL-deficient genotypes. Intr, intronic region; Prom, promoter region. SNPs were added on the basis of chromosome position.

**Figure 2:**
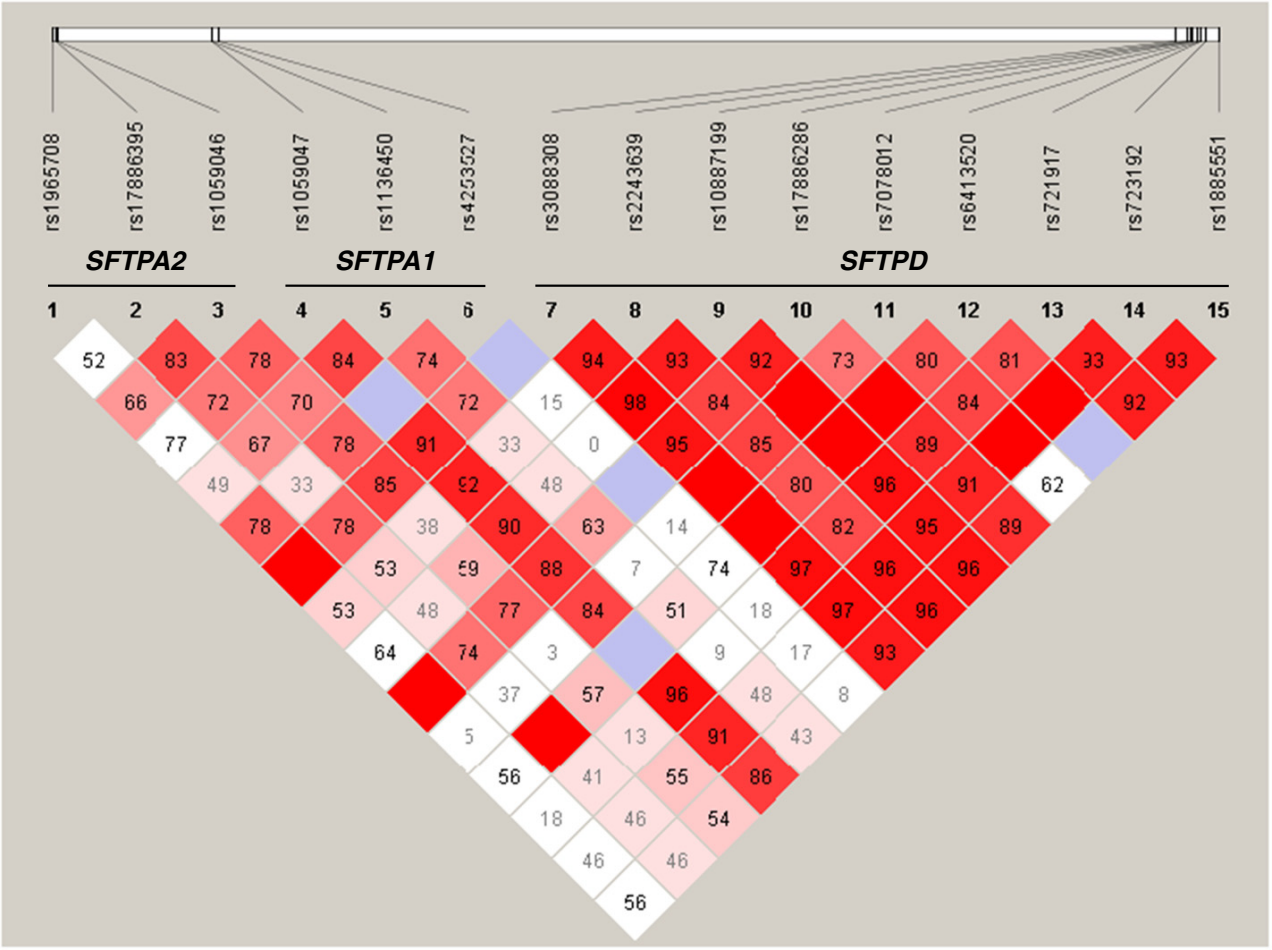
**Linkage disequilibrium (D’) among genetic polymorphisms of surfactant proteins in general Spanish population (*N*** **= 687).** LD plots for pair wise Dʹ between markers and Dʹ values are indicated in percentages within squares in the LD plot. Strong LD is indicated by dark gray/red, whereas light gray/pink and white indicate uninformative and low confidence values, respectively.

When severity of infection was evaluated in hospitalized patients (n = 70) (Table 3), we observed that two missense variants at *SFTPA2* (rs1965708-*C* and rs1059046-*A*) were significantly associated with the need for mechanical ventilation (MV) and with development of ARF and ARDS. Most of these associations remained significant after multivariate analysis adjusted for age, gender, risk factors, and secondary infection (either bacteremia or secondary bacterial pneumonia). The alleles rs1965708-*C* and rs1059046-*A* were overrepresented in patients requiring MV (*P* = 0.003; OR, 2.43; and *P* = 0.016; OR, 3.71, respectively), and in those who developed ARF (*P* = 0.006; OR, 4.09; and *P* = 0.005; OR, 3.70, respectively) or ARDS (*P* = 0.006; OR, 17.68; and *P* = 0.016; OR, 3.71, respectively). The variant rs1965708-*C* was also associated with septic shock (*P* = 0.007; OR, 17.16). The *SFTPA1* allele rs1136450-G was also associated, although to a lower extent, with the development of ARF (*P* = 0.038; OR, 2.64) and the use of MV (*P* = 0.040; OR, 2.55); these associations may be secondary to the existence of LD between *SFTPA1* and *SFTPA2* variants (Figure 2).

**Table 3:** **Severity of H1N1pdm infection in hospitalized patients regarding missense single-nucleotide polymorphisms of *SFTPA2 *** **and *SFTPA1***

					**Statistical significance**			
					***P***^**a**^	***P***^**b**^	***P***^**a**^	***P***^**b**^
					**OR (95% CI)**	**OR (95% CI)**	**OR (95% CI)**	**OR (95% CI)**
**Polymorphism**		**Genotype frequencies**			**Genotype comparisons**		**Allele comparisons**	
**rs1965708 (Q223K)**		***CC***	***CA***	***AA***	***CC vs CA + AA***		***C vs A***	
ARF	48	33 (0.69)	15 (0.31)	0 (0.00)	0.027	0.018	0.006	0.006
No ARF	22	9 (0.41)	10 (0.46)	3 (0.14)	3.18 (1.12-9.01)	5.40 (1.34-21.71)	3.09 (1.35-7.04)	4.09 (1.5-11.13)
ARDS	16	14 (0.88)	2 (0.13)	0 (0.00)	0.018^c^	0.017	0.014	0.006
No ARDS	54	28 (0.52)	23 (0.43)	3 (0.06)	6.45 (1.35-31.25)	7.81 (1.45-42.05)	5.51 (1.24-24.51)	17.68 (2.25-138.57)
Shock	16	14 (0.88)	2 (0.13)	0 (0.00)	0.018^c^	0.018	0.014	0.007
No shock	54	28 (0.52)	23 (0.43)	3 (0.06)	6.45 (1.35-31.25)	7.64 (1.41-41.44)	5.51 (1.24-24.51)	17.16 (2.17-135.70)
MV	24	20 (0.83)	4 (0.17)	0 (0.00)	0.004	0.007	0.004	0.003
No MV	46	22 (0.48)	21 (0.46)	3 (0.07)	5.46 (1.61-18.47)	18.60 (2.23-155.24)	4.57 (1.49-13.97)	12.78 (2.39-68.42)
ICU^e^	30	23 (0.77)	7 (0.23)	0 (0.00)	0.014	0.019	0.010	0.031
No ICU^e^	40	19 (0.48)	18 (0.45)	3 (0.08)	3.64 (1.27-10.42)	2.98 (1.19-7.46)	3.25 (1.29-8.16)	2.43 (1.09-5.45)
**rs1059046 (T9N)**		***AA***	***AC***	***CC***	***AA vs AC + CC***		***A vs C***	
ARF	48	20 (0.42)	24 (0.50)	4 (0.08)	0.020	0.011	0.017	0.005
No ARF	22	3 (0.14)	14 (0.64)	5 (0.23)	4.52 (1.18-17.24)	8.02 (1.61-39.98)	2.40 (1.16-4.98)	3.70 (1.48-9.30)
ARDS	16	9 (0.56)	6 (0.38)	1 (0.06)	0.023	0.014	-	0.016
No ARDS	54	14 (0.26)	32 (0.59)	8 (0.15)	3.68 (1.15-11.77)	5.47 (1.42-21.13)	-	3.71 (1.28-10.72)
MV	24	11 (0.46)	12 (0.50)	1 (0.04)	-	0.039	-	0.016
No MV	46	12 (0.26)	26 (0.57)	8 (0.17)	-	3.59 (1.07-12.10)	-	2.97 (1.22-7.19)
ICU^e^	30	14 (0.47)	14 (0.47)	2 (0.07)	0.033	-	0.036	-
No ICU^e^	40	9 (0.23)	24 (0.60)	7 (0.18)	3.01 (1.07-8.48)	-	2.11 (1.04-4.27)	-
**rs1136450 (L50V)**		***GG***	***GC***	***CC***	***CC vs GC + GG***		***G vs C***	
ARF	48	21 (0.44)	25 (0.52)	2 (0.04)	0.028^c^	0.019	0.024	0.038
No ARF	22	5 (0.23)	12 (0.55)	5 (0.23)	6,77 (1.20-38.22)	19.80 (1.64-238.70)	2.31 (1.11-4.81)	2.60 (1.05-6.42)
MV	24	11 (0.46)	13 (0.54)	0 (0.00)	0.044	-	-	0.040
No MV	46	15 (0.33)	24 (0.52)	7 (0.15)	1.62 (1.33-1.96)	-	-	2.55 (1.04-6.22)

^a^*P* value for the bivariate comparison calculated with the χ^2^ test. ^b^*P* value for the multivariate analysis calculated with binary logistic regression, including the variables age, gender, risk factors, secondary bacterial pneumonia, and bacteremia. ^c^ap value by Fisher Exact test. ^d^ip value for the multivariate analysis calculated with binary logistic regression, including the variables age, gender, risk factors, and secondary bacterial pneumonia (bacteremia variable was excluded because it shows a co-lineal relation with the variable mechanical ventilation). ^e^Patients who required (ICU) or did not require (No ICU) ICU admission, *P* value for the multivariate analysis calculated with conditional logistic regression stratified by hospital of origin, including the co variables age, gender, risk factors, secondary bacterial pneumonia, and bacteremia. Only those comparisons with *P* < 0.05 and significant odds ratios were included. OR (95% CI), Odds ratio (95% confidence interval); ARF, acute respiratory failure; ARDS, acute respiratory distress syndrome; MV, mechanical ventilation; ICU, intensive care unit.

When haplotypes, based on combinations of missense SNP, were analyzed (Table 4), the *SFTPA2* haplotype *1A*^*0*^ was found to be associated with the need for MV and with the development of ARF and ARDS in hospitalized patients. These differences were found to be independent of age, gender, risk factors, and secondary infection (either bacteremia or secondary bacterial pneumonia) in multivariate analysis: *P* = 0.003 (data in Table 4), OR 3.73; *P* = 0.022; OR, 2.81; and *P* = 0.016; OR, 3.41, for the need for MV, ARF, and ARDS comparisons, respectively. By contrast, the *SFTPA2* haplotype *1A*^*1*^ was found to be protective in a dominant model against the requirement for MV and the development of ARF and ARDS in the multivariate analysis (*P* = 0.015; OR, 0.16; *P* = 0.005; OR, 0.21; and *P* = 0.021; OR, 0.12).

**Table 4:** **Severity of H1N1pdm infection in hospitalized patients regarding haplotypes of *SFTPA2***

					**Statistical significance**			
					***P***^**a**^	***P***^**b**^	***P***^**a**^	***P***^**b**^
					**OR (95% CI)**	**OR (95% CI)**	**OR (95% CI)**	**OR (95% CI)**
**Haplotype**		**Diplotype frequencies**			**Diplotype comparisons**		**Haplotype comparisons**	
***1A***^***0***^ ***(AGC)***		***1A***^***0***^ ***/1A***^***0***^	***1A***^***0***^**/rest**	**Rest/rest**	***1A***^***0***^ ***/1A***^***0***^ ***vs 1A***^***0***^**/rest + rest/rest**		***1A***^***0***^ ***vs*** **rest**	
ARF	48	16 (0.33)	27 (0.56)	5 (0.10)	0.031	0.045	0.023	0.022
No ARF	22	2 (0.09)	14 (0.64)	6 (0.27)	5.00 (1.04-24.10)	6.38 (1.04-38.97)	2.30 (1.11-4.76)	2.81 (1.16-6.77)
ARDS	16	8 (0.50)	7 (0.44)	1 (0.06)	0.020^c^	0.013	0.029	0.016
No ARDS	54	10 (0.19)	34 (0.63)	10 (0.19)	4,40.(1.33-14.56)	5.44 (1.43-20.77)	2.56 (1.08-6.02)	3.41 (1.26-9.22)
MV	24	11 (0.46)	12 (0.50)	1 (0.04)	0.005	0.004	0.007	0.003
No MV	46	7 (0.15)	29 (0.63)	10 (0.22)	4.72 (1.51-14.69)	7.03 (1.83-26.95)	2.78 (1.31-5.83)	3.73 (1.55-9.00)
***1A***^***1***^ ***(CGA)***		***1A/1A***^***1***^	***1A***^***1***^**/rest**	**rest/rest**	***1A***^***1***^***/1A***^***1***^ **+** ***1A***^***1***^**/rest*vs*rest/rest**		***1A***^***1***^ ***vs*** **rest**	
ARF	48	0 (0.00)	11 (0.23)	37 (0.77)	0.009	0.0017	0.004	0.005
No ARF	22	2 (0.09)	10 (0.46)	10 (0.46)	0.25 (0.08-0.73)	0.08 (0.02-0.39)	0.23 (0.11-0.68)	0.21 (0.07-0.62)
ARDS	16	0 (0.00)	2 (0.12)	14 (0.88)	-	0.040	-	0.021
No ARDS	54	2 (0.04)	19 (0.35)	33 (0.61)	-	0.16 (0.03- 0.92)	-	0.12 (0.02-0.73)
MV	24	0 (0.00)	4 (0.17)	20 (0.83)	0.037	0.019	0.034	0.015
No MV	46	2 (0.04)	17 (0.37)	27 (0.59)	0.28 (0.08-0.97)	0.08 (0.01-0.66)	0.31 (0.1-0.96)	0.16 (0.04-0.70)
ICU^e^	30	0 (0.00)	4 (0.13)	26 (0.87)	0.003	0.015	0.003	0.029
No ICU^e^	40	2 (0.05)	17 (0.43)	21 (0.53)	0.17 (0.05-0.58)	0.25 (0.08-0.76)	0.20 (0.07-0.62)	0.32 (0.11-0.89)

Only those comparisons with *P* < 0.05 and significant odds ratio were included. OR (95% CI), odds ratio (95% confidence interval); ARF, acute respiratory failure; ARDS, acute respiratory distress syndrome; MV, mechanical ventilation; ICU, intensive care unit.

^a^*P* value for the bivariate comparison calculated with the χ^2^ test. ^b^ + - + value for the multivariate analysis calculated with binary logistic regression, including the variables age, gender, risk factors, secondary bacterial pneumonia, and bacteremia. ^c^*P* value by Fisher Exact test. ^d^*P* value for the multivariate analysis calculated with binary logistic regression, including the variables age, gender, risk factors, and secondary bacterial pneumonia (bacteremia variable was excluded because it shows a co-lineal relation with the variable mechanical ventilation). ^e^Patients who required (ICU) or did not require (No ICU) ICU admission, *P* value for the multivariate analysis calculated with conditional logistic regression stratified by hospital of origin, including the covariables age, gender, risk factors, secondary bacterial pneumonia, and bacteremia. Conventional haplotypes at *SFTPA2* were identified on the basis of combinations of the polymorphism rs1059046 (T9N), rs17886395 (A91P), and rs1965708 (Q223K). “Rest” refers to the other haplotypes for each comparison.

**Figure 3:**
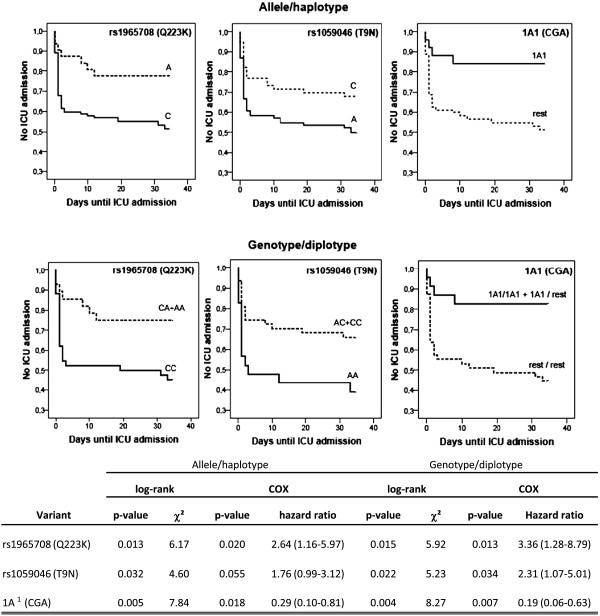
**Kaplan-Meier estimation of days until ICU admission in hospitalized H1N1pdm-infected patients according to*SFTPA2*variants.** Only those comparisons with *P* < 0.05 were included. Solid curves represent the most frequent variant under study, and the dotted curves, the rest of variants. Significance levels calculated by means of log-rank test and Cox regression stratified by hospital of origin and adjusted for the variables age, gender, risk factors, secondary bacterial pneumonia and bacteremia are shown at the bottom of the figure. HR (95% CI), hazard ratio (95% confidence interval).

As an additional measurement of severity, we analyzed the need for ICU admission in hospitalized patients. The variants rs1965708-*C* and rs1059046-*A* were associated with a higher rate of ICU admission, although only rs1965708-*C* remained significant after conditional logistic regression adjustment for the previously mentioned variables and stratified by hospital of origin (*P* = 0.031; OR, 2.43) (Table 3). The same analysis remained significant at *SFTPA2* haplotype *1A*^*1*^ in a dominant model (*P* = 0.029; OR, 0.32) (Table 4). It is noteworthy that Kaplan-Meier analysis and the log-rank test showed that the variants rs1965708-*C* and rs1059046-*A* were associated with a significantly shorter time to ICU admission in hospitalized patients (Figure 3). The same analysis also showed that ICU admission was less frequently required among patients with the haplotype *1A*^*1*^, and this difference was readily detected in the first days after hospitalization. The effect of these variants at the time of ICU admission remained significant in Cox regression.

**Figure 4:**
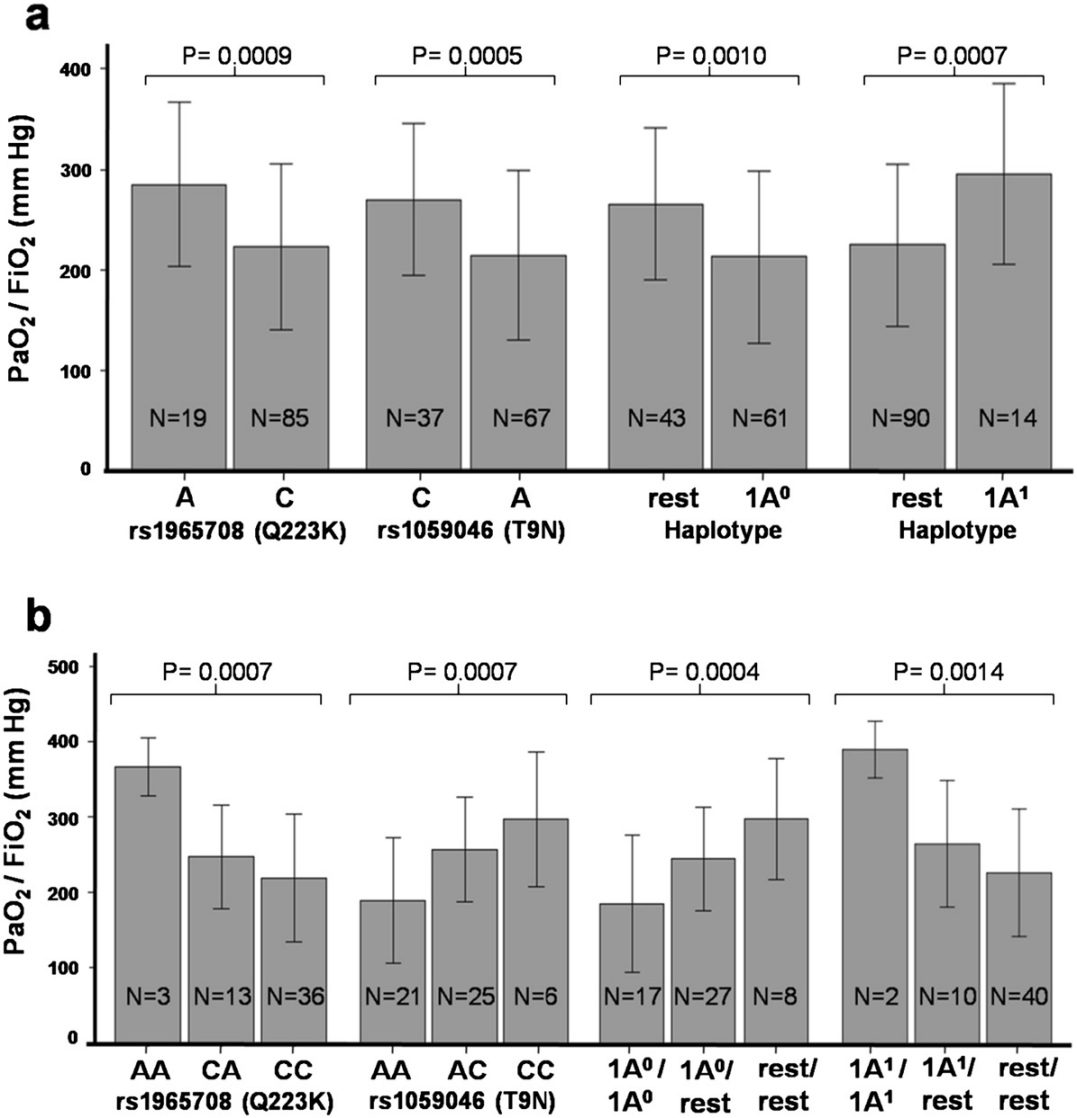
**Ratio of oxygen arterial pressure to oxygen inspiratory fraction (PaO**_**2**_**/FiO**_**2**_**) with regard to*SFTPA2*genetic variants*.*** PaO_2_/FiO_2_ with regard to alleles and haplotypes **(a)** as well as genotypes and diplotypes **(b)** of *SFTPA2* in hospitalized patients with H1N1 pandemic 2009 influenza A virus infection. Each bar represents the mean ± SD. *P* values were calculated with a regression lineal model, including the variables age, gender, risk factors, secondary bacterial pneumonia, and bacteremia.

We finally evaluated the influence of genetic variants of *SFTPA1* and *SFTPA2* on respiratory gas exchange in 52 hospitalized patients. *SFTPA2* alleles (rs1965708-*C* and rs1059046-*A)* and haplotypes (*1A*^*0*^) predisposing to increased severity were found to be associated with significantly lower PaO_2_/FiO_2_ ratios, independent of age, gender, risk factors, secondary bacterial pneumonia, and bacteremia (Figure 4). Interestingly, the effect of these alleles or haplotypes was found to be dependent on the number of alleles present in a genotype or diplotype: *P* = 0.0007, *P* = 0.0007 and *P* = 0.0004 for rs1965708-*C,* rs1059046-*A,* and *1A*^*0*^ respectively. The *1A*^*1*^ haplotype, which was associated with lesser severity, was also associated with higher PaO_2_/FiO_2_ ratios (*P* = 0.0007), and this effect was also found to be dose-dependent in a linear regression model adjusted for the same independent variables (*P* = 0.0014) (Figure 4).

## Discussion

Glycosylation of hemagglutinins may be an important mechanism by which IAV can evade recognition by antibodies in an immune population. By contrast, glycosylation of hemagglutinins is important in determining sensitivity of IAV to recognition by collectins [7,27]. SP-D and MBL bind to mannose-rich glycans on the hemagglutinins and neuraminidase glycoproteins of IAV, agglutinating viral particles and inhibiting infectivity and neuraminidase activity [7]. Among the known human collectins, SP-D has the strongest *in vitro* capability of aggregating and neutralizing activity of IAV [7,12,27]. Hemagglutinins from all available strains of pandemic influenza viruses show significantly lower glycosylation sites compared with seasonal strains; and pandemic viruses, particularly H1N1pdm, were found to be resistant to the antiviral activities of SP-D, MBL, and the pentraxin PTX3 [7,13,27]. We have not observed any association with severity when deficient-, low-, or high-MBL producer genotypes and *SFTPD* SNPs or haplotypes were compared (data not shown). Interestingly, SP-A binding to hemagglutinins and SP-A-dependent IAV neutralization *in vitro* are not influenced by the extent of hemagglutinins glycosylation. SP-A is slightly more effective than SP-D at neutralizing nonglycosylated IAV strains, and it neutralizes IAV strains resistant to SP-D [12].

Accordingly, our results point toward a role of *SFTPA2* SNPs and haplotypes, particularly haplotypes *1A*^*0*^ and *1A*^*1*^, in the severity of H1N1pdm infection. The rationale for such an association seems to be the involvement of *SFTPA2* variants in gas-exchange parameters, which, in the case of pulmonary IAV infection, is largely dependent on IAV-induced lung inflammation.

Besides its role in IAV neutralization, the hydrophilic SP-A and -D have been shown to have an antiinflammatory function. Binding of the carbohydrate-binding recognition domains (CRDs) to signal-inhibitory regulatory protein α (SIRPα) on alveolar macrophages suppresses NF-κB activation and inflammation, allowing the healthy lung to remain in a quiescent state. SP-A and SP-D can also inhibit inflammation, through the CRD, blocking Toll-like receptors 2 and 4. Pulmonary clearance of IAV was reduced, and pulmonary inflammation and severity were increased in *Sftpa*-/-mice compared with wild-type mice [10,11]. SP-A was also found to disturb the host inflammatory response to IAV infection in mouse models without directly influencing viral growth and spread, and even without demonstrable viral binding, at least when the virus was resistant to neutralization by SP-D [9]. Insufficient amounts of surfactant, particularly SP-A, have been observed in prematurely born infants with respiratory distress syndrome (pRDS). The haplotype *1A*^*0*^ or *SFTPA1-SFTPA2* haplotypes containing *1A*^*0*^ have been repeatedly associated with the development of pRDS, whereas SP haplotypes containing *1A*^*1*^ were found to be protective against that condition [5,15]. These findings parallel those observed in our study, suggesting that *1A*^*0*^ and *1A*^*1*^ might influence the inflammatory response and the severity of H1N1pdm infection without binding to IAV.

Human SP-A1 and –A2 have similar *in vitro* hemagglutination-inhibition activity of IAV strains exhibiting non- or poorly glycosylated hemagglutinins heads [14]. However, in all functional assays, SP-A2 is more active than SP-A1 [5,15]. So it is not surprising that among all the genetic variants of collectins analyzed in our study, only a few alleles and haplotypes of *SFTPA2* were associated with H1N1pdm severity in hospitalized patients. Furthermore, the effect of the *SFTPA2* variants on the need for ICU admission was detected in the first few days of hospitalization, as would be expected for components of the innate immunity involved in inflammation and defense against IAV infection.

The significance of the functional differences between variants at *SFTPA1* and *SFTPA2* is poorly understood [5,15]. The variant rs1965708 produces an amino-acid change at residue 223 (Q223K), located in the CRD of SP-A2, and might directly influence the binding properties to either IAV or the antiinflammatory receptor SIRPα. Residue 9 (rs1059046, T9N) is located in the signal peptide, and it is unknown whether this variant may affect the protein. It is, however, worth noting that haplotypes *1A*^*0*^ and *1A*^*1*^ differ precisely at residues 9 and 223. A role of SNPs at regulatory regions in haplotypes *1A*^*0*^ and *1A*^*1*^ in translation and/or RNA stability of *SFTPA2* cannot be ruled out [5,15]*.* Interestingly, SP-A2 1A^1^ variants were shown to have a lower activity for enhancement of TNF-α in macrophage-derived THP-1 cells than other variants, such as 1A and 1A^0^, particularly after oxidative stress [28,29]. These data suggest that the protective effect of the SP-A2 1A^1^ variant in the severity of H1N1pdm infection could be due to its lower proinflammatory activity.

Irrespective of the causal SNP(s), our data suggest that the haplotype *1A*^*0*^ of *SFTPA2* is associated with a higher severity after H1N1pdm infection, whereas the *SFTPA2* haplotype *1A*^*1*^ was associated with a dominant protective effect against severe forms of H1N1pdm infection.

We acknowledge several limitations of our study. First, the overrepresentation of hospitalized patients, could bias us to analyze susceptibility to H1N1pdm infection. In addition, our control group could be considered a representative sample from the Spanish population rather than a representation of non-H1N1pdm-infected individuals.

Second, our study is underpowered to detect the role of some variants on severity of H1N1pdm infection. Nevertheless, considering odds ratios and a significance level of 5%, the power of the associations of rs1965708-*C* with the development of ARDS and septic shock was 84%, and more than 88% for the need for MV. In the haplotype analysis, statistical power was 88.56% for the effect of the haplotype *1A*^*1*^ on ICU admission (90.62% for the effect in a dominant model). No correction for multiple comparisons was performed in these comparisons, but, as expected by *in vivo* and *in vitro* studies among the human collectins, only SP-A would be expected to influence H1N1pdm infectivity, and significant associations were repeatedly observed with several clinical phenotypes. Furthermore, the observed associations were found to be independent of age, gender, risk factors predisposing to severe H1N1 infection, and development of either secondary bacterial pneumonia or bacteremia. Variants of *SFTPA2* were clearly associated with functional parameters of gas exchange, underscoring their role in the severity of H1N1pdm-induced lung injury.

Third, criteria for ICU admission may vary between different centers. To avoid these differences, multivariate and Cox regression analysis to evaluate the need for ICU admission were stratified by hospital of origin.

## Conclusion

Our study suggests that variants at *SFTPA2* influence the severity of H1N1pdm infection in hospitalized patients. The potential of collectins as therapeutic agents for the treatment of IAV-mediated disease is now being explored [7,30]. In *Sftpa*-/-and *Sftpd*-/-mice, intratracheally administered SP-A or SP-D can restore microbial clearance and inflammation [5], and exogenous surfactant preparations containing the hydrophobic SP-B and -C are widely used for replacement therapies in pRDS. The data from our study, together with a better knowledge of the functional significance of the genetic variability at *SFTPA2* on IAV-associated disease, could pave the way for a potential treatment with haplotype-specific (*1A*^*1*^) SP-A2 for patients with the most severe forms of the disease in future IAV pandemics.

## Key messages

• Genetic variation in the *SFTPA2* gene influences the outcome of patients infected with the 2009 pandemic H1N1 influenza A virus.

• Data from our study may help to identify patients at higher risk of severe pandemic (nonglycosylated) IAV infection, who may eventually benefit from more-personalized and targeted therapies.

## Abbreviations

ARDS: Acute respiratory distress syndrome; ARF: acute respiratory failure; BMI: body mass index; CEIC: Comité Ético de Investigación Clínica (Clinical Research Ethics Committee); CRD: carbohydrate recognition domain; FiO_2_: fraction of inspired oxygen; H1N1pdm: virus influenza A H1N1 pandemic; HIV: human immunodeficiency virus; HR: hazard ratio; IAV: influenza A virus; ICU: intensive care unit; MBL: mannose-binding lectin; MODS: multiorgan dysfunction syndrome; MV: mechanical ventilation; NF-κB: nuclear factor kappa B; OR: odds ratio; PaO_2_: partial pressure of oxygen; pRDS: respiratory distress syndrome in prematurely born infant; PVP: primary viral pneumonia; SaO_2_: arterial oxygen saturation; SIRPα: signal inhibitory regulatory protein α; SNP: single-nucleotide polymorphism; SP: surfactant protein.

## Competing interests

The author(s) declare that they have no competing interests.

## Authors’ contributions

EHR: acquisition, analysis, and interpretation of molecular genetic data, and statistical analysis and collaboration in the writing of the manuscript. MLR: acquisition, analysis, and interpretation of molecular genetic data and critical review of the manuscript. JJRH: acquisition, analysis, and interpretation of clinical data and critical review of the manuscript. JPH: acquisition, analysis, and interpretation of clinical data and critical review of the manuscript. LB: acquisition, analysis, and interpretation of clinical data and critical review of the manuscript. EL: acquisition, analysis, and interpretation of clinical data and critical review of the manuscript. JB: acquisition, analysis, and interpretation of clinical data and critical review of the manuscript. MCPG: acquisition, analysis, and interpretation of H1N1pdm infection data and critical review of the manuscript. MIGL: molecular genetic data acquisition, analysis, and interpretation and critical review of the manuscript. YF: molecular genetic data acquisition and critical review of the manuscript. VMB: acquisition, analysis, and interpretation of H1N1pdm infection data and critical review of the manuscript. MM: acquisition, analysis, and interpretation of clinical data and critical review of the manuscript. JMF: acquisition, analysis, and interpretation of clinical data and critical review of the manuscript. LS: acquisition, analysis, and interpretation of clinical data and critical review of the manuscript. CV: acquisition, analysis, and interpretation of H1N1pdm infection data and critical review of the manuscript. OR: acquisition, analysis, and interpretation of clinical data and critical review of the manuscript. MB: acquisition, analysis, and interpretation of clinical data and critical review of the manuscript. JA: acquisition, analysis, and interpretation of clinical data and critical review of the manuscript. ELG: acquisition, analysis, and interpretation of clinical data and critical review of the manuscript. JSV: acquisition, analysis, and interpretation of clinical data and collaboration in the writing of the manuscript. FRC: financial support, acquisition, analysis, and interpretation of clinical data and collaboration in the writing of the manuscript. CRG: coordination, conception and design, financial support, and manuscript writing. All authors read and approved the final version of the manuscript.

## Authors’ information

Jordi Solé-Violán and Felipe Rodríguez de Castro should be regarded as co-second last senior authors.
